# Dichlorido(2,9-dieth­oxy-1,10-phenanthroline-κ^2^ *N*,*N*′)zinc(II)

**DOI:** 10.1107/S1600536809024490

**Published:** 2009-07-01

**Authors:** Cao-Yuan Niu, Yu-Li Dang, Xin-Sheng Wan, Chun-Hong Kou

**Affiliations:** a College of Sciences, Henan Agricultural University, Zhengzhou 450002, People’s Republic of China

## Abstract

All non-H atoms except for the Cl atoms lie on a mirror plane in the title complex, [ZnCl_2_(C_16_H_16_N_2_O_2_)]. The Zn^II^ ion is coordinated by two N atoms from a bis-chelating 2,9-dieth­oxy-1,10-phenanthroline ligand and two symmetry-related Cl atoms in a distorted tetra­hedral environment. The two Zn—N bond lengths are significantly different from each other and the N—Zn—N angle is acute. In the crystal structure, there are weak but significant π–π stacking inter­actions between phenanthroline rings, with a centroid–centroid distance of 3.764 (1) Å.

## Related literature

For background information, see: Majumder *et al.* (2006 ▶); Bie *et al.* (2006 ▶). For synthetic details, see: Pijper *et al.* (1984 ▶).
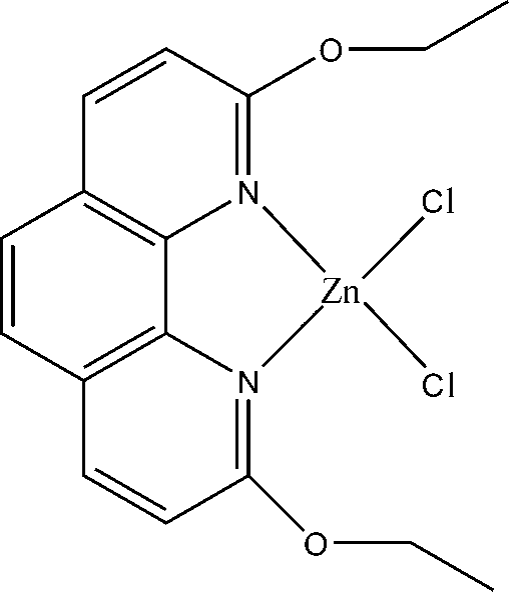


## Experimental

### 

#### Crystal data

[ZnCl_2_(C_16_H_16_N_2_O_2_)]
*M* *_r_* = 404.58
Orthorhombic, 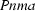

*a* = 13.255 (3) Å
*b* = 7.4403 (15) Å
*c* = 17.874 (4) Å
*V* = 1762.7 (6) Å^3^
*Z* = 4
Mo *K*α radiation
μ = 1.71 mm^−1^
*T* = 291 K
0.20 × 0.18 × 0.17 mm

#### Data collection

Bruker APEX-II CCD diffractometer
Absorption correction: multi-scan (*SADABS*; Sheldrick, 1996 ▶) *T*_min_ = 0.727, *T*_max_ = 0.760
5148 measured reflections
1741 independent reflections
1303 reflections with *I* > 2σ(*I*)
*R*_int_ = 0.052

#### Refinement

*R*[*F* ^2^ > 2σ(*F* ^2^)] = 0.048
*wR*(*F* ^2^) = 0.089
*S* = 1.08
1741 reflections
136 parameters
H-atom parameters constrained
Δρ_max_ = 0.34 e Å^−3^
Δρ_min_ = −0.58 e Å^−3^

### 

Data collection: *SMART* (Siemens, 1996 ▶); cell refinement: *SAINT* (Siemens, 1994 ▶); data reduction: *SAINT*; program(s) used to solve structure: *SHELXS97* (Sheldrick, 2008 ▶); program(s) used to refine structure: *SHELXL97* (Sheldrick, 2008 ▶); molecular graphics: *SHELXL97* and *DIAMOND* (Brandenburg, 2005 ▶); software used to prepare material for publication: *SHELXL97*.

## Supplementary Material

Crystal structure: contains datablocks I, global. DOI: 10.1107/S1600536809024490/lh2850sup1.cif

Structure factors: contains datablocks I. DOI: 10.1107/S1600536809024490/lh2850Isup2.hkl

Additional supplementary materials:  crystallographic information; 3D view; checkCIF report

## Figures and Tables

**Table:** 

Zn1—N1	2.065 (3)
Zn1—N2	2.118 (4)
Zn1—Cl1	2.2022 (10)
Zn1—Cl1^i^	2.2022 (10)

**Table:** 

N1—Zn1—N2	79.43 (13)
N1—Zn1—Cl1	112.53 (5)
N2—Zn1—Cl1	112.90 (4)
N1—Zn1—Cl1^i^	112.53 (5)
N2—Zn1—Cl1^i^	112.90 (4)
Cl1—Zn1—Cl1^i^	119.74 (6)

Symmetry code: (i) 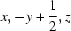.

**Fig. 1.**
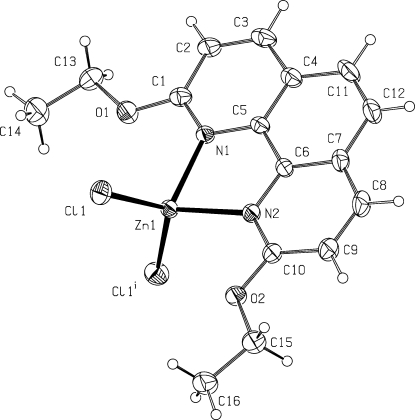
The molecular structure of the title complex, showing the atom-labelling scheme. Displacement ellipsoids are drawn at the 30% probability level and H atoms are shown as small spheres of arbitrary radii. [Symmetry codes: (i) x, -y + 1/2, z.]

**Fig. 2.**
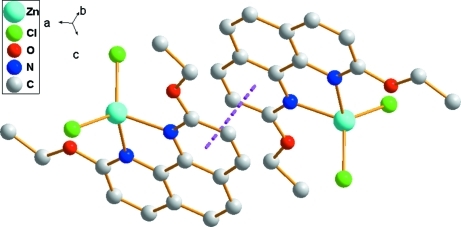
Part of the crystal structure showing a π–π interaction (purple dotted line). All H atoms have been omitted for clarity.

## supplementary crystallographic information

### Comment

The compound 1,10-phenanthroline has been reported as used to synthesize some potential strong luminescent materials with d^10^ metals. It was predicted that the title compound which is composed of a derivative of 1,10-phenanthroline and a d^10 ^metal would possess strong ligand to ligand or metal perturbed ligand to ligand emissions (Majumder *et al.*, 2006; Bie, *et al.*, 2006). The ligand 2,9-Diethoxy-1,10-phenanthroline as a derivative of 1,10-phenanthroline was synthesized at an earlier time and possesses antimycoplasmal activity in the presence of copper (Pijper, *et al.*, 1984).

The title mononuclear zinc(II) complex is shown in Fig. 1. All non-hydrogen atoms, execpt for the Cl atoms, lie on a mirror plane. The Zn^II^ ion is four coordinated by two nitrogen atoms from the 1,10-phenanthroline ring system (N1 and N2) and two chlorine atoms [Cl1, Cl1^i^. Symmetry code: (i) *x*, -*y* + 1/2, *z*], defining a disotorted tetrahedral coordination environment.  In the crystal structure there are weak but significant π–π stacking interactions between phenanthroline rings (Fig. 2) with a centroid-to-centroid distance of 3.764 (1) Å.

### Experimental

The organic ligand 2,9-diethoxy-1,10-phenanthroline was prepared according to the procedure of literature (Pijper, *et al.*, 1984). The slow evaporation of mixture of the ligand (0.024 g, 0.1 mmol) and zinc dichloride (0.014 g, 0.1 mmol) in 30 ml me thanol afforded suitable colourless block crystals in about 7 days (yield 60%).

### Refinement

Carbon-bound H atoms were positioned geometrically and refined using a riding model [C—H = 0.93 Å and *U*_iso_(H) = 1.2 *U*_eq_(C) for aromatic H atoms; C—H = 0.97 Å and *U*_iso_(H) = 1.2 *U*_eq_(C) for methylene H atoms; C—H = 0.96 Å and *U*_iso_(H) = 1.5 *U*_eq_(C) for methyl H atoms;]. The final difference Fourier map had a highest peak at 1.17 Å from atom Zn1 and a deepest hole at 1.04 Å from atom Zn1.

### Crystal data

**Table:**

[ZnCl_2_(C_16_H_16_N_2_O_2_)]	*F*(000) = 824
*M**_r_* = 404.58	*D*_x_ = 1.524 Mg m^−^^3^
Orthorhombic, *P**n**m**a*	Mo *K*α radiation, λ = 0.71073 Å
Hall symbol: -P 2ac 2n	Cell parameters from 398 reflections
*a* = 13.255 (3) Å	θ = 2–25.1°
*b* = 7.4403 (15) Å	µ = 1.71 mm^−^^1^
*c* = 17.874 (4) Å	*T* = 291 K
*V* = 1762.7 (6) Å^3^	Prismatic, colorless
*Z* = 4	0.20 × 0.18 × 0.17 mm

### Data collection

**Table:**

Bruker APEX-II CCD detector diffractometer	1741 independent reflections
Radiation source: fine-focus sealed tube	1303 reflections with *I* > 2σ(*I*)
graphite	*R*_int_ = 0.052
Detector resolution: 0 pixels mm^-1^	θ_max_ = 25.5°, θ_min_ = 1.9°
Oscillation frames scans	*h* = −16→0
Absorption correction: multi-scan (*SADABS*; Sheldrick, 1996)	*k* = −8→8
*T*_min_ = 0.727, *T*_max_ = 0.760	*l* = −21→21
5148 measured reflections	

### Refinement

**Table:**

Refinement on *F*^2^	Primary atom site location: structure-invariant direct methods
Least-squares matrix: full	Secondary atom site location: difference Fourier map
*R*[*F*^2^ > 2σ(*F*^2^)] = 0.048	Hydrogen site location: inferred from neighbouring sites
*wR*(*F*^2^) = 0.089	H-atom parameters constrained
*S* = 1.08	*w* = 1/[σ^2^(*F*_o_^2^) + (0.042*P*)^2^] where *P* = (*F*_o_^2^ + 2*F*_c_^2^)/3
1741 reflections	(Δ/σ)_max_ < 0.001
136 parameters	Δρ_max_ = 0.34 e Å^−^^3^
0 restraints	Δρ_min_ = −0.58 e Å^−^^3^

### Special details

**Table:**

Geometry. All e.s.d.'s (except the e.s.d. in the dihedral angle between two l.s. planes) are estimated using the full covariance matrix. The cell e.s.d.'s are taken into account individually in the estimation of e.s.d.'s in distances, angles and torsion angles; correlations between e.s.d.'s in cell parameters are only used when they are defined by crystal symmetry. An approximate (isotropic) treatment of cell e.s.d.'s is used for estimating e.s.d.'s involving l.s. planes.
Refinement. Refinement of *F*^2^ against ALL reflections. The weighted *R*-factor *wR* and goodness of fit *S* are based on *F*^2^, conventional *R*-factors *R* are based on *F*, with *F* set to zero for negative *F*^2^. The threshold expression of *F*^2^ > σ(*F*^2^) is used only for calculating *R*-factors(gt) *etc*. and is not relevant to the choice of reflections for refinement. *R*-factors based on *F*^2^ are statistically about twice as large as those based on *F*, and *R*- factors based on ALL data will be even larger.

### Fractional atomic coordinates and isotropic or equivalent isotropic displacement parameters (Å^2^)

**Table:**

	*x*	*y*	*z*	*U*_iso_*/*U*_eq_	Occ. (<1)
Zn1	−0.76441 (4)	0.2500	0.40645 (3)	0.03224 (18)	
Cl1	−0.72431 (7)	−0.00599 (12)	0.35222 (5)	0.0513 (3)	
O1	−0.9753 (2)	0.2500	0.33493 (19)	0.0549 (10)	
O2	−0.5679 (2)	0.2500	0.51182 (18)	0.0441 (9)	
N1	−0.9098 (2)	0.2500	0.4479 (2)	0.0326 (9)	
N2	−0.7355 (2)	0.2500	0.5230 (2)	0.0330 (9)	
C1	−0.9938 (3)	0.2500	0.4084 (3)	0.0409 (12)	
C2	−1.0897 (3)	0.2500	0.4426 (3)	0.0464 (14)	
H2A	−1.1480	0.2500	0.4136	0.056*	
C3	−1.0957 (4)	0.2500	0.5183 (3)	0.0512 (15)	
H3A	−1.1585	0.2500	0.5415	0.061*	
C4	−1.0068 (4)	0.2500	0.5625 (3)	0.0425 (13)	
C5	−0.9155 (3)	0.2500	0.5242 (3)	0.0325 (11)	
C6	−0.8217 (3)	0.2500	0.5640 (3)	0.0327 (11)	
C7	−0.8228 (4)	0.2500	0.6423 (3)	0.0411 (12)	
C8	−0.7279 (4)	0.2500	0.6775 (3)	0.0530 (14)	
H8A	−0.7244	0.2500	0.7295	0.064*	
C9	−0.6412 (4)	0.2500	0.6367 (3)	0.0489 (15)	
H9A	−0.5787	0.2500	0.6604	0.059*	
C10	−0.6474 (3)	0.2500	0.5576 (3)	0.0382 (13)	
C11	−1.0056 (4)	0.2500	0.6427 (3)	0.0576 (16)	
H11A	−1.0663	0.2500	0.6689	0.069*	
C12	−0.9174 (4)	0.2500	0.6808 (3)	0.0537 (15)	
H12A	−0.9183	0.2500	0.7329	0.064*	
C13	−1.0535 (4)	0.2500	0.2795 (3)	0.0527 (15)	
H13A	−1.0956	0.3561	0.2843	0.063*	0.50
H13B	−1.0956	0.1439	0.2843	0.063*	0.50
C14	−0.9988 (4)	0.2500	0.2065 (3)	0.081 (2)	
H14A	−1.0468	0.2500	0.1663	0.122*	
H14B	−0.9572	0.1446	0.2032	0.122*	0.50
H14C	−0.9572	0.3554	0.2032	0.122*	0.50
C15	−0.4675 (3)	0.2500	0.5431 (3)	0.0578 (17)	
H15A	−0.4576	0.1442	0.5740	0.069*	0.50
H15B	−0.4576	0.3558	0.5740	0.069*	0.50
C16	−0.3943 (4)	0.2500	0.4794 (3)	0.0608 (17)	
H16A	−0.3266	0.2500	0.4987	0.091*	
H16B	−0.4046	0.3554	0.4494	0.091*	0.50
H16C	−0.4046	0.1446	0.4494	0.091*	0.50

### Atomic displacement parameters (Å^2^)

**Table:**

	*U*^11^	*U*^22^	*U*^33^	*U*^12^	*U*^13^	*U*^23^
Zn1	0.0278 (3)	0.0404 (3)	0.0285 (3)	0.000	0.0007 (2)	0.000
Cl1	0.0576 (6)	0.0440 (6)	0.0523 (6)	0.0023 (5)	0.0032 (5)	−0.0099 (5)
O1	0.0288 (18)	0.102 (3)	0.034 (2)	0.000	−0.0083 (16)	0.000
O2	0.0246 (17)	0.070 (3)	0.038 (2)	0.000	−0.0068 (15)	0.000
N1	0.024 (2)	0.042 (3)	0.031 (2)	0.000	−0.0012 (17)	0.000
N2	0.030 (2)	0.040 (2)	0.029 (2)	0.000	−0.0015 (19)	0.000
C1	0.030 (2)	0.045 (3)	0.048 (3)	0.000	0.000 (3)	0.000
C2	0.023 (2)	0.064 (4)	0.052 (4)	0.000	−0.006 (2)	0.000
C3	0.030 (3)	0.058 (4)	0.065 (4)	0.000	0.013 (3)	0.000
C4	0.036 (3)	0.047 (3)	0.044 (3)	0.000	0.009 (2)	0.000
C5	0.030 (2)	0.030 (3)	0.037 (3)	0.000	0.008 (2)	0.000
C6	0.034 (3)	0.035 (3)	0.029 (3)	0.000	0.005 (2)	0.000
C7	0.052 (3)	0.044 (3)	0.027 (3)	0.000	0.002 (2)	0.000
C8	0.062 (4)	0.072 (4)	0.025 (3)	0.000	−0.009 (3)	0.000
C9	0.040 (3)	0.072 (4)	0.034 (3)	0.000	−0.009 (3)	0.000
C10	0.033 (3)	0.049 (4)	0.033 (3)	0.000	−0.006 (2)	0.000
C11	0.048 (3)	0.076 (5)	0.048 (4)	0.000	0.025 (3)	0.000
C12	0.054 (3)	0.077 (5)	0.029 (3)	0.000	0.008 (3)	0.000
C13	0.038 (3)	0.069 (4)	0.050 (4)	0.000	−0.017 (3)	0.000
C14	0.051 (4)	0.149 (7)	0.044 (4)	0.000	−0.013 (3)	0.000
C15	0.030 (3)	0.096 (5)	0.047 (4)	0.000	−0.011 (3)	0.000
C16	0.034 (3)	0.093 (5)	0.056 (4)	0.000	−0.003 (3)	0.000

### Geometric parameters (Å, °)

**Table:**

Zn1—N1	2.065 (3)	C7—C8	1.406 (7)
Zn1—N2	2.118 (4)	C7—C12	1.431 (7)
Zn1—Cl1	2.2022 (10)	C8—C9	1.362 (7)
Zn1—Cl1^i^	2.2022 (10)	C8—H8A	0.9300
O1—C1	1.336 (6)	C9—C10	1.415 (6)
O1—C13	1.434 (5)	C9—H9A	0.9300
O2—C10	1.335 (5)	C11—C12	1.353 (7)
O2—C15	1.443 (5)	C11—H11A	0.9300
N1—C1	1.318 (5)	C12—H12A	0.9300
N1—C5	1.366 (6)	C13—C14	1.493 (7)
N2—C10	1.322 (5)	C13—H13A	0.9700
N2—C6	1.358 (5)	C13—H13B	0.9700
C1—C2	1.410 (6)	C14—H14A	0.9600
C2—C3	1.356 (7)	C14—H14B	0.9600
C2—H2A	0.9300	C14—H14C	0.9600
C3—C4	1.418 (7)	C15—C16	1.496 (7)
C3—H3A	0.9300	C15—H15A	0.9700
C4—C5	1.390 (6)	C15—H15B	0.9700
C4—C11	1.434 (7)	C16—H16A	0.9600
C5—C6	1.433 (6)	C16—H16B	0.9600
C6—C7	1.400 (6)	C16—H16C	0.9600
			
N1—Zn1—N2	79.43 (13)	C7—C8—H8A	119.5
N1—Zn1—Cl1	112.53 (5)	C8—C9—C10	119.1 (5)
N2—Zn1—Cl1	112.90 (4)	C8—C9—H9A	120.5
N1—Zn1—Cl1^i^	112.53 (5)	C10—C9—H9A	120.5
N2—Zn1—Cl1^i^	112.90 (4)	N2—C10—O2	114.2 (4)
Cl1—Zn1—Cl1^i^	119.74 (6)	N2—C10—C9	121.3 (4)
C1—O1—C13	123.1 (4)	O2—C10—C9	124.5 (4)
C10—O2—C15	119.3 (4)	C12—C11—C4	120.8 (5)
C1—N1—C5	119.2 (4)	C12—C11—H11A	119.6
C1—N1—Zn1	126.6 (3)	C4—C11—H11A	119.6
C5—N1—Zn1	114.2 (3)	C11—C12—C7	121.0 (5)
C10—N2—C6	119.4 (4)	C11—C12—H12A	119.5
C10—N2—Zn1	128.4 (3)	C7—C12—H12A	119.5
C6—N2—Zn1	112.3 (3)	O1—C13—C14	104.6 (4)
N1—C1—O1	111.8 (4)	O1—C13—H13A	110.8
N1—C1—C2	122.0 (5)	C14—C13—H13A	110.8
O1—C1—C2	126.3 (4)	O1—C13—H13B	110.8
C3—C2—C1	119.0 (5)	C14—C13—H13B	110.8
C3—C2—H2A	120.5	H13A—C13—H13B	108.9
C1—C2—H2A	120.5	C13—C14—H14A	109.5
C2—C3—C4	120.5 (5)	C13—C14—H14B	109.5
C2—C3—H3A	119.7	H14A—C14—H14B	109.5
C4—C3—H3A	119.7	C13—C14—H14C	109.5
C5—C4—C3	116.6 (5)	H14A—C14—H14C	109.5
C5—C4—C11	118.9 (5)	H14B—C14—H14C	109.5
C3—C4—C11	124.5 (5)	O2—C15—C16	107.6 (4)
N1—C5—C4	122.7 (4)	O2—C15—H15A	110.2
N1—C5—C6	116.6 (4)	C16—C15—H15A	110.2
C4—C5—C6	120.7 (5)	O2—C15—H15B	110.2
N2—C6—C7	123.3 (4)	C16—C15—H15B	110.2
N2—C6—C5	117.5 (4)	H15A—C15—H15B	108.5
C7—C6—C5	119.2 (4)	C15—C16—H16A	109.5
C6—C7—C8	116.0 (4)	C15—C16—H16B	109.5
C6—C7—C12	119.3 (5)	H16A—C16—H16B	109.5
C8—C7—C12	124.6 (5)	C15—C16—H16C	109.5
C9—C8—C7	121.0 (4)	H16A—C16—H16C	109.5
C9—C8—H8A	119.5	H16B—C16—H16C	109.5
			
N2—Zn1—N1—C1	180.0	C10—N2—C6—C7	0.000 (1)
Cl1—Zn1—N1—C1	−69.44 (5)	Zn1—N2—C6—C7	180.0
Cl1^i^—Zn1—N1—C1	69.44 (5)	C10—N2—C6—C5	180.0
N2—Zn1—N1—C5	0.0	Zn1—N2—C6—C5	0.0
Cl1—Zn1—N1—C5	110.56 (5)	N1—C5—C6—N2	0.0
Cl1^i^—Zn1—N1—C5	−110.56 (5)	C4—C5—C6—N2	180.0
N1—Zn1—N2—C10	180.0	N1—C5—C6—C7	180.000 (1)
Cl1—Zn1—N2—C10	69.87 (5)	C4—C5—C6—C7	0.000 (1)
Cl1^i^—Zn1—N2—C10	−69.87 (5)	N2—C6—C7—C8	0.000 (1)
N1—Zn1—N2—C6	0.0	C5—C6—C7—C8	180.000 (1)
Cl1—Zn1—N2—C6	−110.13 (5)	N2—C6—C7—C12	180.000 (1)
Cl1^i^—Zn1—N2—C6	110.13 (5)	C5—C6—C7—C12	0.000 (1)
C5—N1—C1—O1	180.0	C6—C7—C8—C9	0.000 (1)
Zn1—N1—C1—O1	0.0	C12—C7—C8—C9	180.000 (1)
C5—N1—C1—C2	0.0	C7—C8—C9—C10	0.000 (1)
Zn1—N1—C1—C2	180.0	C6—N2—C10—O2	180.0
C13—O1—C1—N1	180.0	Zn1—N2—C10—O2	0.0
C13—O1—C1—C2	0.0	C6—N2—C10—C9	0.000 (1)
N1—C1—C2—C3	0.000 (1)	Zn1—N2—C10—C9	180.0
O1—C1—C2—C3	180.0	C15—O2—C10—N2	180.0
C1—C2—C3—C4	0.000 (1)	C15—O2—C10—C9	0.000 (1)
C2—C3—C4—C5	0.000 (1)	C8—C9—C10—N2	0.000 (1)
C2—C3—C4—C11	180.000 (1)	C8—C9—C10—O2	180.000 (1)
C1—N1—C5—C4	0.000 (1)	C5—C4—C11—C12	0.000 (1)
Zn1—N1—C5—C4	180.0	C3—C4—C11—C12	180.000 (1)
C1—N1—C5—C6	180.0	C4—C11—C12—C7	0.000 (2)
Zn1—N1—C5—C6	0.0	C6—C7—C12—C11	0.000 (2)
C3—C4—C5—N1	0.0	C8—C7—C12—C11	180.000 (1)
C11—C4—C5—N1	180.0	C1—O1—C13—C14	180.0
C3—C4—C5—C6	180.0	C10—O2—C15—C16	180.0
C11—C4—C5—C6	0.000 (1)		

Symmetry codes: (i) *x*, −*y*+1/2, *z*.
